# Bilateral Reflex Fluctuations during Rhythmic Movement of Remote Limb Pairs

**DOI:** 10.3389/fnhum.2017.00355

**Published:** 2017-07-05

**Authors:** Rinaldo A. Mezzarane, Tsuyoshi Nakajima, E. Paul Zehr

**Affiliations:** ^1^Laboratory of Signal Processing and Motor Control, College of Physical Education, University of BrasíliaBrasília, Brazil; ^2^Rehabilitation Neuroscience Laboratory, School of Exercise Science, Physical, and Health Education, University of VictoriaVictoria, BC, Canada; ^3^Department of Integrative Physiology, Kyorin University School of MedicineTokyo, Japan; ^4^Human Discovery Science, International Collaboration on Repair Discoveries (ICORD)Vancouver, BC, Canada; ^5^Centre for Biomedical Research, University of VictoriaVictoria, BC, Canada; ^6^Division of Medical Sciences, University of VictoriaVictoria, BC, Canada

**Keywords:** cross-covariance, variability, H-reflex, human, spinal cord

## Abstract

The modulation of spinal cord excitability during rhythmic limb movement reflects the neuronal coordination underlying actions of the arms and legs. Integration of network activity in the spinal cord can be assessed by reflex variability between the limbs, an approach so far very little studied. The present work addresses this question by eliciting Hoffmann (H-) reflexes in both limbs to assess if common drive onto bilateral pools of motoneurons influence spinal cord excitability simultaneously or with a delay between sides. A cross-covariance (CCV) sequence between reflexes in both arms or legs was evaluated under conditions providing common drive bilaterally through voluntary muscle contraction and/or rhythmic movement of the remote limbs. For H-reflexes in the flexor carpi radialis (FCR) muscle, either contraction of the FCR or leg cycling induced significant reduction in the amplitude of the peak at the zero lag in the CCV sequence, indicating independent variations in spinal excitability between both sides. In contrast, for H-reflexes in the soleus (SO) muscle, arm cycling revealed no reduction in the amplitude of the peak in the CCV sequence at the zero lag. This suggests a more independent control of the arms compared with the legs. These results provide new insights into the organization of human limb control in rhythmic activity and the behavior of bilateral reflex fluctuations under different motor tasks. From a functional standpoint, changes in the co-variability might reflect dynamic adjustments in reflex excitability that are subsumed under more global control features during locomotion.

## Introduction

Changes in the excitability of reflex pathways while remote limbs are performing voluntary rhythmic movements has been extensively described (Loadman and Zehr, 2007; Zehr et al., 2007; de Ruiter et al., 2010; Mezzarane et al., 2011). The most likely explanation is the output from neuronal oscillators (presumably located in different segments of the spinal cord) can regulate the reflex excitability. Suppression in the amplitude of the soleus (SO) H-reflex (a homologous of the stretch reflex), observed during arm cycling at an ergometer, was shown to be partially due to an increase of presynaptic inhibition (Frigon et al., 2004). In a later investigation, Nakajima et al. (2013) reported that H-reflex from flexor carpi radialis (FCR) was suppressed by leg rhythmic movement through the same regulatory mechanism. Thus, the inhibitory interneurons that synapse on Ia terminals within the muscle afferent pathway are the main targets of the influences from rhythmic movements of the remote limb. Additionally, these results are evidence of a reciprocal organization between networks involved in rhythmic movements within the spinal cord, i.e., cervical networks affect spinal cord excitability in the lower limbs, and vice-versa, by similar mechanism of reflex modulation. Modulation induced by rhythmic movement of one arm on reflexes in the legs have also been documented (Loadman and Zehr, 2007), but the effect from contralateral arm is weaker than the ipsilateral one.

Presynaptic inhibition can decrease monosynaptic reflex variability, as indicated by the results obtained in cats (Rudomin and Dutton, 1967), but no study has so far addressed the presynaptic mechanisms behind reflex fluctuations in humans. However, the significant correlation between background electromyographic (EMG) activity and H-reflex amplitude indicates that fluctuations in membrane potential of the motoneurons are partially responsible for reflex variability in humans (Funase and Miles, 1999). The study of reflex variability is useful to gain insights into the dynamic influence of synaptic inputs from different origins (e.g., supraspinal, peripheral or propriospinal) onto motoneurons and synaptic terminals interposed in spinal cord pathways. It is generally accepted that fluctuations in reflex excitability arise from both pre- and post-synaptic origins (Rudomin and Dutton, 1967; Gossard et al., 1994; Funase and Miles, 1999), and these changes in reflex excitability could affect the coordinated bilateral muscle activation during locomotion. Therefore, it is also of interest to compare the reflex variability between both limbs.

Previous investigations during rhythmic movement were typically conducted either with reflexes evoked in an independent manner (no time-linkage between reflexes) or without focus on the temporal processes contributing to variability of reflex amplitude. Thus, the characteristics of reflex variability during rhythmic movement (e.g., the coefficient of variation (CV), auto and cross covariance) remain relatively unexplored. With the cross covariance (CCV) technique, it is possible to detect concomitant bilateral fluctuations in reflex excitability and reveal temporal linkages between the legs. Significant CCV peak at the zero lag was found between sequences of H-reflexes elicited simultaneously in the SO muscle at rest in about 50% of the subjects examined (Mezzarane and Kohn, 2002). This means that, despite elements of random changes in reflex excitability, with pre- and post-synaptic origins (Rudomin and Dutton, 1967; Gossard et al., 1994; Funase and Miles, 1999), reflex amplitudes co-vary between the legs.

The proportion of significant peaks at the zero-lag found in Mezzarane and Kohn’s study (Mezzarane and Kohn, 2002) indicates a relatively weak common (or correlated) influence to both lower limbs in the resting state. The source of this influence is unknown, but it was hypothesized to originate from descending pathways, such as those from the corticospinal or reticulospinal tracts (Mezzarane and Kohn, 2002). In a latter study, sequences of bilateral monosynaptic reflexes obtained in cats with transection at spinal cord L1 produced significant peaks at the zero-lag in the CCV (Manjarrez et al., 2005). The observed bilateral fluctuations were interpreted as the action of commissural interneurons within laminae III-IV, suggesting spinal contributions (Manjarrez et al., 2005).

Prior studies on bilateral reflex fluctuations were performed in either anesthetized animal preparations or in humans at rest. Evaluation of effects from an “active” common source remains unavailable. Here we focused on bilateral spinal cord processes achieved by the performance of different motor tasks with presumed differential effects on pool excitability. For instance, arm cycling induces a suppression in the amplitude of the SO H-reflexes from both legs (Frigon et al., 2004; Loadman and Zehr, 2007; de Ruiter et al., 2010), yet voluntary contraction leads to an increase in their amplitude (Crenna and Frigo, 1987; Schieppati, 1987; Burke et al., 1989; Funase and Miles, 1999; Misiaszek, 2003).

In the present work, we sought to describe the simultaneous reflex variability during the performance of different motor tasks. Changes in reflex excitability during homonymous muscle contraction and/or rhythmic movement of a remote limb are expected to occur bilaterally. We hypothesized each manipulation would induce higher amplitude peaks at the zero-lag in the CCV sequences, as compared to resting. It was predicted that both rhythmic arm cycling and voluntary SO muscle activation performed at the same time would produce an even higher peak amplitude in the CCV as compared to the performance of one task alone. Finally, a possible reciprocal organization of any bilateral common effects, i.e., correlation of reflex fluctuations between both arms during FCR activation, was assessed at rest and during leg cycling.

## Materials and Methods

### Participants

Twenty volunteers aged 28.3 ± 5.2 years (means ± SD) participated with written informed consent under a protocol (Ethics Protocol Number: 07-480-04d) approved by the Human Research Ethics Committee at the University of Victoria and in accordance with the guidelines in the Declaration of Helsinki. The protocol involved two experiments to assess the role of rhythmic limb activity: (1) Experiment 1 with legs stationary and arm cycling; and (2) Experiment 2 with arms stationary and leg cycling.

### Procedures

#### Methodology Common to Both Experiments

**Figure 1:**
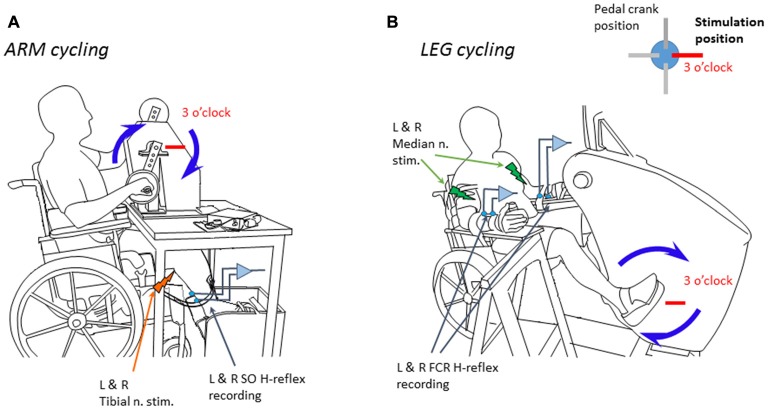
Schema showing the position of the subject during the experiments and the ergometers. **(A)** Experiment 1 during arm cycling. **(B)** Experiment 2 during leg cycling. Blue arrows indicate the clockwise movement of the limbs at 1 Hz. The stimuli were delivered every time the right hand/foot crossed the 3’oclock position (indicated by a red horizontal bar). L & R mean, respectively, left and right nerve stimulation or electromyographic (EMG) recording. Adapted from Nakajima et al. (2013) with permission.

SO muscle H-reflexes from both legs were evoked with a percutaneous electrical squared pulse (1 ms duration) applied to the tibial posterior nerve at the popliteal fossa of both legs. A constant current stimulator Grass S88 (Grass Instruments, AstroMed) connected in series with a SIU5 isolator and a CCU1 constant current unit delivered the stimulus. FCR muscle H-reflexes were obtained by an electrical stimulus (1 ms duration) applied to the median nerve through bipolar surface electrodes placed just proximal to the medial epicondyle of the humerus (Zehr et al., 2007; Nakajima et al., 2013). The H-reflexes had amplitudes of ~20%–30% of the maximal direct response (Mmax) from either SO or FCR muscle. Maximal voluntary contraction (MVC) at the ankle and wrist to obtain EMG recordings of SO or FCR muscles were performed before the beginning of the experiment. Subjects performed either arm or leg cycling on an ergometer at ~1 Hz during ~8.5 min with the help of a visual feedback displayed on an oscilloscope screen. As in prior studies (Loadman and Zehr, 2007; Mezzarane et al., 2014), the full movement cycle of the ergometers was divided into 12 parts corresponding to hours on a clock face. The H-reflex was evoked at every cycle of the ergometer, i.e., every time that the arm crossed the 3 o’clock position the H-reflexes were evoked in both SOs simultaneously (Figure 1A). Similarly, in other manipulation, every time that the leg crossed the 3 o’clock position the H-reflexes were evoked in both FCRs simultaneously (Figure 1B).

A total of 510 stimuli was simultaneously delivered to both legs in each of the four conditions in the following order: (1) static, no movement (St) of the arms; (2) arm cycling at 1 Hz (Cy); (3) no arm movement with bilateral homonymous muscle (SO) isometric contraction (StCo); (4) arm cycling with bilateral SO contraction (CyCo). In the experiment to obtain H-reflex in the FCR, the same procedure was adopted, but with leg cycling instead or arm cycling. The conditions were: (1) static, no movement (St) of the legs; (2) leg cycling at 1 Hz (Cy); (3) no leg movement with bilateral homonymous muscle (FCR) isometric contraction (StCo); and (4) leg cycling with bilateral FCR contraction (CyCo). For conditions 3 and 4, subjects maintained a consistent tonic contraction (10% of MVC) of either the SO or FCR muscles from both legs/arms using visual feedback of the EMG level displayed on a computer screen.

##### Experiment 1: influences of arm cycling on reflexes in the legs

Participants (*n* = 12) sat in a custom-adapted armchair that minimized extraneous movement. Subjects performed arm cycling on a customized ergometer (Loadman and Zehr, 2007; Mezzarane et al., 2014; Figure 1A). The angles of knee and ankle joint were at 120° and 110°, respectively. Surface EMG was recorded bilaterally from five muscles: SO; tibialis anterior (TA); vastus lateralis (VL), biceps femoris (BF) and anterior deltoid (AD).

##### Experiment 2: influences of leg cycling on reflexes in the arms

Twelve participants (eight new participants and four from Experiment 1) participated in this experiment. Subjects performed bilateral leg cycling on an instrumented cycle ergometer (SciFit Pro II Systems, Tulsa, UK; Nakajima et al., 2013; Figure 1B). The arms were fixed to plates with elbow angle at 120°. A brace was worn to restrict movement about both wrist joints (Figure 1B). Surface EMG was recorded bilaterally from five muscles: FCR; extensor carpi radialis (ECR); AD, biceps brachialis (BB) and SO.

### Data Acquisition

Disposable 1 cm Ag–AgCl surface electrodes (Thought Technologies, Edmonton, AB, Canada) with inter-electrode distance of 2 cm were used for all EMG recordings. The ground electrode was placed on bony landmarks near the target muscle. The skin over the belly of each muscle was prepared by using alcohol swabs. The EMG signals were sampled at 2.5 kHz with a 12-bit A/D converter connected to a computer running custom-written Lab View software (National Instruments, Austin, TX, USA). The signals were band-pass filtered (10–1000 Hz) by a Grass P511 amplifier (Grass Instruments, AstroMed). The sweeps of EMG were 100 ms duration with a 20 ms pre-stimulus period. All data were stored for offline analysis using the Matlab software (Mathworks, Natick, MA, USA).

### Data Analysis

Peak-to-peak amplitudes of H-reflexes and M-waves along with the root mean square (RMS) values of the background EMG (corresponding to 20 ms pre-stimulus period) were calculated. The H-reflex amplitudes obtained in all conditions were normalized to Mmax.

Each H-reflex sequence with 510 responses per limb had its first 10 responses discarded to eliminate the initial transient due mostly to homosynaptic depression. Thus, the 500 reflex responses were at the depression plateau (Mezzarane and Kohn, 2002). The CV, the ratio between standard deviation and the mean, was calculated for the remaining 500 responses. Each sequence of 500 H-reflexes was detrended by the subtraction of the best regression line fit to the time series.

The CCV sequence was evaluated by using a Matlab routine and was normalized to give value 1 for fully correlated train of reflex responses. The CCV estimated from sequences that show dependence between samples might present a significant peak, even when the two series are not correlated. Therefore, in order to whiten the sequence (making the H-reflexes independent from each other), an auto-regressive (AR) model was adjusted to each sequence. The sequence of H-reflexes was then filtered by the corresponding inverse filter obtained from the AR coefficients. The order of each AR model was chosen (in the range from 1 to 20) to minimize the AIC criterion (Brockwell and Davis, 1991) and a 95% confidence interval could be calculated as being $1.96/\sqrt{N}$, where *N* is the number of samples in the H-reflex sequence (in our case *N* = 500; Brockwell and Davis, 1991). Therefore, in the present work, considering *N* = 500, the critical value is 0.0877. Samples in the CCV above or below the critical value indicate a correlation between the two series at the corresponding lag.

A three-way analysis of variance (ANOVA) with repeated measures was used to detect main effects and interactions of the variables H-reflex, M-wave, CV and background EMG (RMS values) among the factors “activity” (contraction × no contraction), “movement” (cycling × no cycling) and “side” (right × left limb). A two-way ANOVA with repeated measures was conducted to detect main effects and interactions in the amplitude of the CCV peak at the zero-lag among the factors “activity” and “movement”. These statistical analysis were performed through the statistical package SPSS (Chicago, IL, USA). The significance level of all tests was set at *p* < 0.05.

## Results

### Voluntary Contraction and Cycling Effects

**Figure 2:**
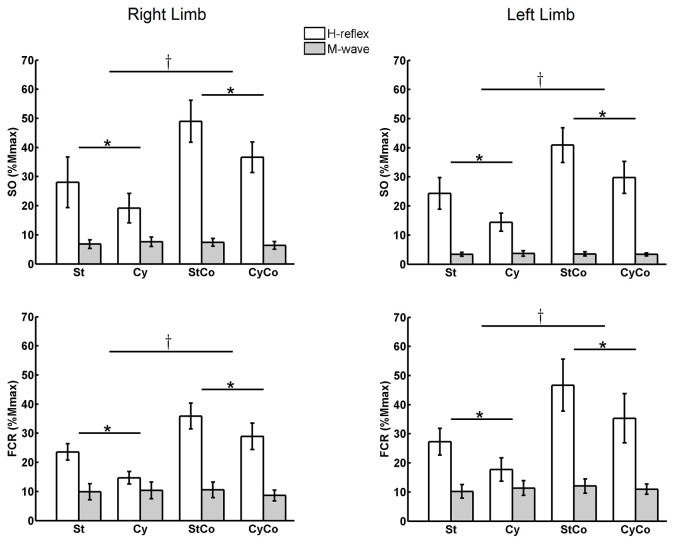
Mean amplitude of H-reflexes and M-waves averaged across the series of 500 responses for both muscles (soleus (SO) and flexor carpi radialis (FCR)) from both legs. Three-way analysis of variance (ANOVA) detected significant main effects in H-reflex amplitudes for the factors “movement” and “activity” (indicated with * and † symbols, respectively; *p* < 0.05). No differences were detected in the M-waves. St, static; Cy, cycling; StCo, static during tonic voluntary contraction of the target muscles; CyCo, cycling condition during tonic voluntary contraction of the target muscles. The vertical lines indicate SEM.

During static (St) condition, the H-reflexes amplitudes from both SO and FCR muscles of the right and left sides were between 20 and 30% of the Mmax (Figure 2). The three-way ANOVA of repeated measures detected a main effect for “activity”, i.e., voluntary homonymous isometric muscle contraction induced significant increase in H-reflex amplitudes (normalized by Mmax) for both SO (*F*_(1,22)_ = 43.881, *p* < 0.001) and FCR (*F*_(1,22)_ = 20.295, *p* < 0.001) muscles (Figure 2). During bilateral voluntary SO contraction, without movement of the arms (condition static with contraction: StCo), the H-reflex of the right SO showed a mean increase of 74.9% from the control condition (St), and the H-reflex of the left SO an increase of 68.3% (% of the control condition). During arm cycling with simultaneous bilateral SO contraction (condition CyCo), there was an average increase in the right SO H-reflex amplitude of 91.0%, as compared to the respective control condition (cycling without contraction: Cy). A 107.1% increase in H-reflex amplitude was observed for the left SO in the same condition. These changes in the normalized reflex amplitudes are depicted in Figure 2. The FCR muscle showed similar pattern of reflex modulation in response to voluntary contraction for both cycling and no cycling (at rest) conditions. At rest, bilateral contraction of the FCR (condition StCo) increased the H-reflex from right and left FCRs, respectively, by 52.1% and 71.2% of the St condition. In CyCo condition, the FCR H-reflexes increased their amplitudes to 96.9% and 99.4% from Cy condition for right and left FCRs, respectively (Figure 2).

The three-way ANOVA also detected significant main effect for the factor “movement”. H-reflex suppression was observed in the SO muscle during arm cycling (*F*_(1,22)_ = 19.161, *p* < 0.001) and in the FCR muscle during leg cycling (*F*_(1,22)_ = 30.566, *p* < 0.001). During arm cycling with no SO voluntary contraction (condition Cy), there was a 31.6% and 40.8% suppression in the SO H-reflex amplitude, respectively for right and left legs, as compared to control condition (St). With SO contraction (condition CyCo), the H-reflex from right and left SO was suppressed during arm cycling, respectively, by 25.3% and 27.1% from their control values (StCo). Again, the same pattern was observed for the FCR muscle: there was a suppression of 37.7% and 35.0%, respectively in the right and left FCR H-reflexes, during Cy as compared to St. In condition CyCo, leg cycling induced a suppression of 19.3% and 24.3%, in right and left FCRs respectively, as compared to the respective control condition (StCo).

No main effect was detected for the factor “side” in both the SO (*F*_(1,22)_ = 0.591, *p* = 0.450) and the FCR muscles (*F*_(1,22)_ = 0.805, *p* = 0.379), suggesting that the effects of cycling and contraction did not differ between right and left sides. Additionally, the modulation of reflex amplitudes in response to constant voluntary isometric muscle contraction did not differ between conditions (cycling or rest). Similarly, the effect of cycling of the remote limb was not significantly different between both contraction (StCo) and no contraction (St) conditions. These observation are confirmed by the three-way ANOVA that did not detect any interaction between the factors “movement” and “activity” for both SO (*F*_(1,22)_ = 1.981, *p* = 0.173) and FCR (*F*_(1,22)_ = 0.001, *p* = 0.973) muscles, which suggests that the effect of cycling is not related to muscle activation (Figure 2).

**Figure 3:**
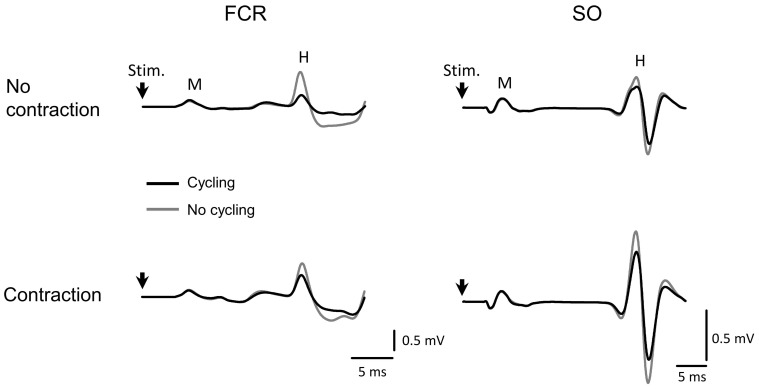
Representative data from one subject showing the average of 500 H-reflex sweeps recorded from the SO and FCR muscles. The upper traces in gray are the averaged sweep evaluated at rest from FCR (left) and SO (right) muscles. The black sweeps are the average sweeps evaluated during cycling. The lower traces represent the same as in the upper traces during contraction at 10% maximal voluntary contraction (MVC) of the homonymous muscle. The vertical arrow indicates the moment of stimulus delivery (the artifact was erased for better visualization). Stim, moment of stimulus delivery (stimulus artifact); M, M-wave; H, H-reflex. Vertical and horizontal lines at the bottom right indicate the calibration.

No significant differences or interactions were found for M-waves between most conditions, indicating an efficient control of stimulation. One exception was a main effect for the factor “side” in the SO muscle (*F*_(1,22)_ = 8.428, *p* = 0.008), showing that M-waves were lower in the left leg as compared to the right, but this difference has no implication for the control of the experiment. These results are displayed in Figure 2. Representative data from one subject can be seen in Figure 3 for both SO and FCR muscles.

### Bilateral Simultaneous Reflex Fluctuations

**Figure 4:**
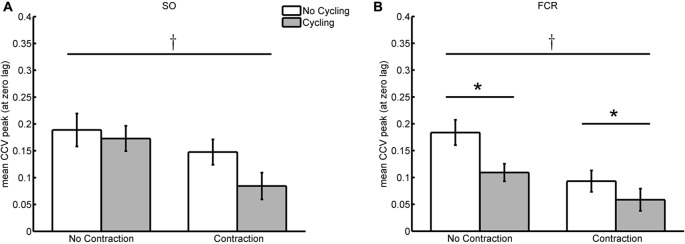
Averaged values of cross-covariance (CCV) peak at zero-lag during cycling/no cycling of the remote limb and contraction/no contraction of the homonymous muscle. **(A,B)** Depict, respectively, the values from the SO and FCR muscle pairs. The cross indicates significant difference (*p* < 0.05) between contraction and no contraction conditions, whereas the asterisk represent significant difference between cycling and no cycling conditions.

The peak values at the zero-lag in the sequence of CCV between reflexes from SO muscles of both legs was, on the average, lower during bilateral SO contraction regardless of the cycling condition (at rest or during cycling) as detected by the two-way ANOVA of repeated measures (*F*_(1,11)_ = 5.646; *p* = 0.037; Figure 4A). The decrease in CCV peak amplitude during SO contraction was 21.8% from its control value with the arms at rest (compare the white bars of Figure 4) and 51.2% compared to the condition with arm cycling (gray bars of Figure 4A). No main affect was observed for the factor “movement”, indicating that arm cycling did not change the CCV peak at the zero-lag irrespective of the muscle activation (*F*_(1,11)_ = 4.220; *p* = 0.065). Despite the effect of contraction inducing a 51.2% of inhibition in CCV values at the zero-lag during cycling (as compared to the reduction of 21.8% at rest), no interaction between factors “movement” and “activity” was detected (*F*_(1,11)_ = 1.051; *p* = 0.327).

Leg cycling induced significant reduction in reflex synchronism between both arms, and for both contraction and no-contraction conditions (*F*_(1,11)_ = 7.883; *p* = 0.017; Figure 4B). The reduction in the CCV peak at the zero-lag observed during leg cycling with FCR voluntary contraction (condition CyCo) was 37.1% from the control condition (StCo) and without contraction (Cy) the reduction was 40.6% from St condition. Significant reduction was also detected for muscle activation irrespective of the movement of the legs (*F*_(1,11)_ = 12.847; *p* = 0.004). During leg cycling, FCR contraction (condition CyCo) reduced in 46.4% the CCV peak at the zero-lag from its respective control condition (Cy). When the legs were at rest, contraction of the FCR (StCo) reduced the CCV to 49.3% from its control value (static, no contraction—St; Figure 4B).

No interaction between the tasks (movement of the legs and FCR voluntary activity) was detected (*F*_(1,11)_ = 1.293; *p* = 0.280). Both conditions induced significant reduction in bilateral reflex fluctuations, but the lack of significant interaction between tasks indicates that the effect of leg cycling and FCR contraction on the co-variability of bilateral reflex excitability did not differ.

**Figure 5:**
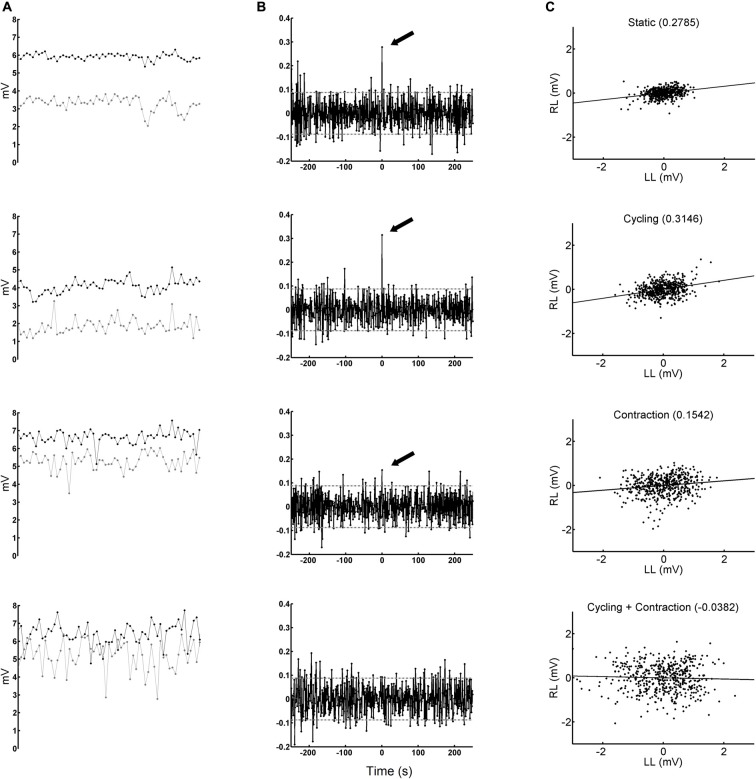
Peak-to-peak amplitudes of H-reflexes obtained from one representative subject. Left-most column **(A)** black and gray circles show the amplitude of H-reflexes obtained from right and left legs, respectively, during the delivery of a sequence of 500 stimuli at 1 Hz. The panels from top to bottom represent conditions static (St), cycling (Cy), contraction (StCo) and cycling + contraction (CyCo), respectively. The panels display 60 amplitudes obtained in each second (the total period shown is 1 min). Middle column **(B)** CCV evaluated from the sequence of H-reflex amplitudes (the same ones of the left column) after being whitened. The significant peaks at zero-lag are indicated with a black arrow (*p* < 0.05). Note the absence of significant peak at zero lag in condition CyCo. The abscissa is calibrated in seconds. The horizontal gray lines delimit the critical values. Right-most column **(C)** scatter plots of 500 H-reflexes from right (RL) and left legs (LL). The negative values are the result of to the detrend process. On the top of each panel is displayed the Pearson’s product-moment correlation coefficient, which is also indicated with the arrows from the panels in the middle column (except for condition CyCo).

The peak-to-peak amplitude values of the SO H-reflex recorded across 1 min from both legs of a representative subject in all four conditions are shown in Figure 5A. During arm cycling (condition Cy), 11 out of 12 subjects showed statistically significant peak at the zero-lag in the CCV sequence (Table 1), which means that reflex excitability synchronously fluctuated in both legs. The conditions static (St) and cycling (Cy) had 83.3% of significant peaks for SO (Table 1), and 75% and 58.3%, respectively, for FCR (Table 2).

**Table 1:** Peak values of the cross-covariance (CCV) sequence (at the zero-lag) between soleus (SO) muscles in all conditions.

Subject	St	Cy	StCo	CyCo
S1 (M)	−0.0027	0.2053*	0.1583*	−0.0324
S2 (F)	0.2563*	0.1728*	0.0715	0.0406
S3 (M)	0.1835*	0.1316*	0.1575*	0.0507
S4 (M)	0.1865*	0.2311*	0.0077	0.0389
S5 (F)	0.1887*	0.1294*	0.2060*	0.1822*
S6 (M)	0.2153*	0.0292	0.1671*	0.0555
S7 (M)	0.2359*	0.2712*	0.2489*	0.1785*
S8 (F)	0.2905*	0.1630*	0.1881*	0.2492*
S9 (F)	0.3292*	0.2295*	0.2564*	0.0727
S10 (M)	0.2785*	0.3146*	0.1542*	−0.0382
S11 (F)	0.0021	0.0852	0.1486*	0.1182*
S12 (M)	0.1009*	0.1116*	0.0044	0.1038*
Peak (%)	83.3%	83.3%	75.0%	41.7%

*The bottom row shows the percentage occurrences for significant peak proportion for each condition taken across participants. Asterisks indicate significant peak (*p* < 0.05). St, static; Cy, cycling; StCo, contraction; CyCo, cycling + contraction; F, female; M, male*.

**Table 2:** Peak values of the CCV sequence (at the zero-lag) between flexor carpi radialis (FCR) muscles in all conditions.

Subject	St	Cy	StCo	CyCo
S9 (F)	0.0798	0.1771*	0.0741	−0.0036
S10 (M)	0.2578*	0.1346*	0.0915*	0.1073*
S11 (M)	0.1883*	0.0751	−0.007	0.0396
S12 (M)	0.2660*	0.2252*	0.0843	0.0724
S13 (M)	0.2408*	0.0738	0.0871	0.1046*
S14 (F)	0.0844	0.1414*	0.1372*	0.2014*
S15 (M)	0.0955*	0.0963*	0.1834*	0.0253
S16 (F)	0.2356*	0.1229*	0.1010*	0.0040
S17 (F)	0.0704	0.1271*	−0.0431	−0.0274
S18 (M)	0.1575*	0.0470	0.0753	−0.0065
S19 (F)	0.2647*	0.0414	0.1333*	0.0257
S20 (M)	0.2641*	0.0481	0.2007*	0.1601*
Peak (%)	75.0%	58.3%	50.0%	33.3%

*The bottom line indicates the significant peak proportion for each condition. Asterisks indicate significant peak (*p* < 0.05). St, static; Cy, cycling; StCo, contraction; CyCo, cycling + contraction; F, female; M, male*.

The peak was considered significant when it crossed the limit of ±0.0877 (see “Data Analysis” Section), drawn with a horizontal line in the CCV sequences displayed in Figure 5B. Correlation between bilateral fluctuations in reflex amplitudes can be seen by visual inspection in Figure 5A and statistically confirmed by the significant peak that crossed the critical value, indicated by an arrow in the CCV sequences of the whitened reflex values in Figure 5B (except for the condition CyCo in which the peak was not significant).

**Figure 6:**
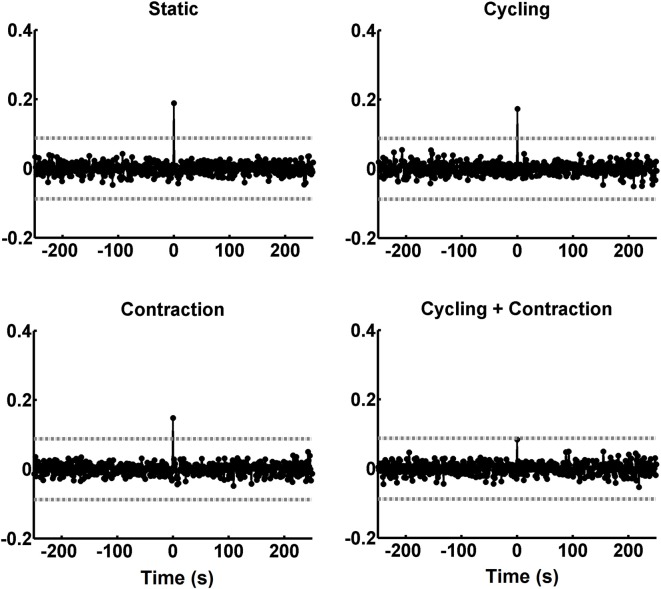
CCV sequences averaged across the 12 subjects. The peaks of the CCV tended to cancel each other along the averaging process, except for the zero lag. The abscissa is calibrated in seconds. The horizontal gray lines indicate the critical values (*p* < 0.05).

Figure 5C shows the scatter plot between reflex amplitudes from the right and left legs after being filtered with the inverse AR coefficients. This explains the differences in amplitude values between Figures 5A,C. The occurrence of negative amplitude values is due to the detrend process on the H-reflex sequence (see “Materials and Methods” Section). The straight line in the graphs of Figure 5C represents the best linear fit to the data and the coefficient of correlation (which coincides with the CCV peak at the zero-lag, indicated by an arrow in Figure 5B) is between brackets on the top of each graph. The plot with data from condition CyCo shows reduced correlation as compared to the remaining conditions. The averaged CCV sequences calculated in all conditions are shown in Figure 6 to highlight the consistency of the zero-lag peak. This confirms that the reflex amplitudes varied in a synchronous way with no time lag between both processes (Figure 6).

**Figure 7:**
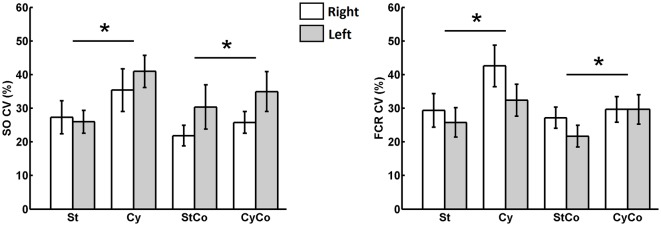
Coefficient of variation (CV) evaluated from the sequence of 500 bilateral H-reflexes from SO (leg) and FCR (arm) muscles. The asterisk indicated significant main effect (*p* < 0.05) of the cycling condition and no main effects of either contraction or sides (left and right).

The graphs in Figure 5C show that the variability of SO muscle H-reflexes increased during rhythmic movement of the arms. To confirm this observation, the relative variability (the ratio between standard deviation and the mean) was calculated for each sequence. The CV evaluated from the sequence of 500 SO H-reflexes was significantly higher during arm cycling (Figure 7) and showed a main effect for the factor “movement” (*F*_(1,22)_ = 9.857, *p* = 0.005). No main effect was observed for the factor “activity” (*F*_(1,22)_ = 1.153, *p* = 0.295) as well as no interaction between factors “movement” and “activity” (*F*_(1,22)_ = 3.790, *p* = 0.064). The same result was found for the CV calculated from the sequence of 500 H-reflexes in the FCR muscle, there was main effect for “movement” (*F*_(1,22)_ = 13.987, *p* = 0.001), with no significant effect for “activity” (*F*_(1,22)_ = 3.151, *p* = 0.09) and no interaction (*F*_(1,22)_ = 1.778, *p* = 0.196). There was no main effect for the factor “side” for both SO (*F*_(1,22)_ = 3.151, *p* = 0.09) and FCR (*F*_(1,22)_ = 1.012, *p* = 0.325), suggesting the effects of cycling were not different between both sides. Taken together, these results suggest that rhythmic movement of remote limbs induced a relative higher variability in H-reflex amplitudes of the stationary target limb/muscle.

### Background EMG Activity

#### Arm Movement

**Figure 8:**
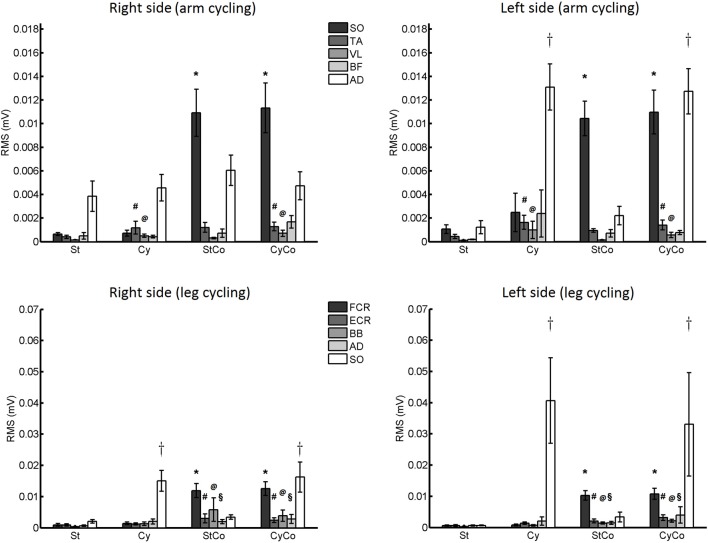
Root mean square (RMS) values of background EMG activity. Upper panels: data from Experiment 1 (arm cycling). Lower panels: data from Experiment 2 (leg cycling). Asterisk indicates significant effect of contraction. The crosses indicate significant effect of cycling. The vastus lateralis (VL) and biceps femoris (BF) muscles showed main effects for arm cycling (# and @, respectively; *p* < 0.05). The extensor carpi radialis (ECR), biceps brachialis (BB) and anterior deltoid (AD) muscles showed main effect for contraction of the FCR muscle (#, @ and §, respectively; *p* < 0.05). The vertical lines are SEM.

A significant main effect for the factor “activity” was revealed by the three-way ANOVA test performed on the RMS values of background EMG recorded from the SO muscle (20 ms before stimulus delivery; *F*_(1,22)_ = 49.069, *p* < 0.001; Figure 8). The AD muscle was more active during arm cycling, which explains the main effect for the factor “movement” (*F*_(1,22)_ = 20.129, *p* < 0.001).

The factor “movement” showed a significant main effect for VL (*F*_(1,22)_ = 6.222, *p* = 0.021) and TA (*F*_(1,22)_ = 7.433, *p* = 0.012) muscles, with a very low muscle activation (around 1 μV and 2 μV, respectively) that does not interfere with the amplitude of the H-reflex. No significant difference or interaction was found for the BF muscle (Figure 8).

#### Leg Movement

The activity of the FCR muscle was higher during voluntary contraction (Figure 8), and a main effect for the factor “activity” was detected in both FCR (*F*_(1,22)_ = 52.571, *p* < 0.001) and ECR muscles (*F*_(1,22)_ = 11.672, *p* = 0.002). The SO muscle was more active during leg cycling, as can be confirmed by the significant main effect of the factor “movement” (*F*_(1,22)_ = 12.259, *p* = 0.002).

Main effects were detected for the factor “activity” in both BB (*F*_(1,22)_ = 4.656, *p* = 0.042) and AD (*F*_(1,22)_ = 5.390, *p* = 0.030) muscles, but again these muscle activities were noticeably low, i.e., lower than 6 μV and 3 μV for BB and AD, respectively (Figure 8).

## Discussion

This article provides evidence of common influences on the reduced synchronism in the variability of reflex excitability of both legs and arms during the performance of different motor tasks. The concomitant fluctuation in spinal cord excitability suggests neural commands from different origins are common to both limbs. Contrary to our initial hypothesis, contraction of homonymous left and right muscles reduced the simultaneous variations in reflex amplitudes of both pairs of limbs (legs and arms). However, cycling movement of the remote limb induced significant reduction in the co-variability between the arms, but not between the legs. This means that the current observed increase in the relative variability (CV) of reflex responses for both pairs of limbs (see Figure 7) cannot account for the reduction in co-variation between the arms during leg cycling (see Figure 4). Thus, actions from suprasegmental structures, spinal cord networks, or afferents from the moving limbs independently influence reflex variability of each side of the spinal cord, but at the same time they can simultaneously influence the two sides of the spinal cord.

Each motor task seems to have a specific influence on reflex co-variability for arms and legs. For instance, the failure to detect reductions in bilateral fluctuation between the legs during the performance of arm cycling may be evidence of specific differences in the limb pairs, i.e., these observations might reflect the action of lumbar spinal cord mechanisms to assure suitable coordination between limbs during locomotion. Conversely, the lack of concomitant reflex fluctuations between the arms, regardless of the motor task, suggests a more independent control of each arm as compared to the legs during locomotion.

### Arm Cycling and Bilateral Reflex Fluctuations in the Legs

Previously, bilateral fluctuations in spinal cord excitability was investigated in subjects at rest with H-reflexes evoked at a fixed 1 s interval (Mezzarane and Kohn, 2002). About half of the subjects showed significant peak in the CCV sequence at the zero-lag, suggesting simultaneous reflex modulation. The non-significant cross-correlation of the other half of the sample could be explained by independent input to both legs. This could also be explained by a process that has “memory” time course shorter than 1 s, however, the averaged auto-covariances estimated for each leg in the study of Mezzarane and Kohn (2002) showed significant dependence between H-reflex amplitudes sampled at intervals higher than 1 s (indeed, up to ~16 s). Similar behavior has been observed in the current study, strong dependence between reflexes evoked with stimulus delivered nearly every 1 s, as can be qualitatively verified in Figure 5A. These results indicate that the processes behind H-reflex fluctuations have memory longer than 1 s, therefore, absence of significant CCV peak at the zero-lag cannot be explained by the possible independence between reflexes sampled with 1 s interval. In this context, Mezzarane and Kohn (2002) hypothesized that descending commands from supraspinal centers (e.g., conveyed by the corticospinal and/or reticulospinal pathways) could be responsible for the concomitant bilateral fluctuations in reflex excitability, since peripheral influence were minimized. In the present work, St condition showed higher mean amplitude of the CCV peak at the zero-lag as compared to the remaining conditions. This result could be ascribed to remote muscle activation due the postural configuration: the subjects were seated and holding an ergometer. Thus, the activities of trunk and neck muscles in static condition could exert some influence on reflex amplitude of the legs (Schieppati, 1987), which can explain the higher amplitude of the CCV peak.

Results of Manjarrez et al. (2005) from a spinalized feline model support the view of a neural source simultaneously affecting the excitability of reflex pathways from both legs. The observation of significant CCV between the monosynaptic reflexes from both sides of the spinal cord persisting after spinalization, indicates a source partially confined in the spinal cord. These bilateral fluctuations only ceased after a longitudinal bisection of the spinal cord disrupting the action of commissural interneurons that probably mediated the synchronous fluctuations in monosynaptic reflex excitability (Manjarrez et al., 2005). A question remained as to what extent the performance of motor tasks would affect the correlation between both sides in humans.

Arm cycling activates oscillatory networks presumably located in the cervical region of the spinal cord that exert influences on reflex excitability of the legs (Loadman and Zehr, 2007; de Ruiter et al., 2010; Dragert and Zehr, 2013; Mezzarane et al., 2014). The lack of significant reduction in the amplitude of the CCV peak at the zero-lag during arm cycling could be accounted for by the signals coming from cervical neuronal oscillators. This neuronal network would preserve the currently observed synchronicity between reflex responses of the legs, possibly through the action of propriospinal interneurons that connect lumbosacral and cervical enlargements as described in the cat (Juvin et al., 2012), and speculated to be responsible for interlimb coordination during locomotion in humans (MacLellan et al., 2013). Experiments with rat have shown that the left-right interaction is mediated by commissural interneurons that are recruited by descending projections from bulbospinal (Cowley et al., 2009) and reticulospinal (Mitchell et al., 2016) tracts. Therefore, the brainstem locomotor commands can regulate the activity of lumbar commissural interneurons that probably contribute to the synchronism of the bilateral monosynaptic reflexes (Manjarrez et al., 2005). These commissural interneurons are also the target from other sources such as group I and II afferents (Jankowska et al., 2009) evidencing the variety of inputs involved in bilateral reflex fluctuations. In this regard, crossed effects within lumbar spinal cord mediated by different classes of afferents, point to the existence of commissural interneurons involved in coordinated actions during locomotion in humans (Cheng et al., 1998; Stubbs et al., 2011).

During voluntary contraction, descending commands from motor cortex to motoneurons probably contributed to decreased simultaneous fluctuations in reflex excitability in both upper and lower limb pairs. A significant correlation of the motor evoked potentials (MEP) from upper limbs, in response to transcranial magnetic stimulation in the motor area of both sides, indicates a synchronization in cortical excitability of both hemispheres (Ellaway et al., 1998). Interestingly, when the subject performs a bilateral voluntary contraction, the correlation between the amplitude of the pairs of MEPs was suppressed (Pearce et al., 2005). As the amplitude of the MEP is associated with cortex excitability, the results found by Pearce et al. (2005) can be interpreted as change in bilateral fluctuations in cortical excitability. Therefore, despite spinal cord mechanisms, reduction in CCV peak at the zero-lag might also reflect independent inputs from the motor cortex to the pools of motoneurons of both sides. It is worth emphasizing, however, that the present experimental protocol is not suitable to identify precisely the sources of the influences that increases or decreases bilateral reflex excitability simultaneously.

Earlier report from cat experiments showed that a presynaptic mechanism can affect reflex variability (Rudomin and Dutton, 1967). In humans, evidence has been provided that the output of the cervical central pattern generator (CPG; activated by arm cycling) modulates lumbar spinal reflex by means of a presynaptic inhibition onto Ia afferent terminal (Frigon et al., 2004). Thus, the presynaptic mechanism of reflex modulation active during arm cycling from CPG-related cervical regions could influence reflex variability of both sides, given that arm cycling modulates reflex amplitude of both legs (Loadman and Zehr, 2007). It is important to point out, however, that the effects of arm cycling are stronger when the ipsilateral arm (to the leg being stimulated) reaches a given position (de Ruiter et al., 2010). This phase dependency induces stronger effects upon the ipsilateral leg and weaker effects to the contralateral one: when the right arm reaches the 3 o’clock position in our experimental paradigm, the left arm is at 9 o’clock position. The neural command coming from cervical oscillators are probably not evenly distributed to both pools of motoneurons. Therefore, differential influence on reflex excitability must be considered in the interpretations of CCV results, i.e., the synchronism in reflex fluctuations between the legs could be more prominent if the signals from arm cycling reached the lumbar spinal cord with similar strengths.

From a functional standpoint, bilateral reflex modulation depends on the phase of the gait cycle and the respective position of the arms. There is a clear phase-dependent reflex modulation and alternated activation between ankle flexors and extensors muscles during walking (Capaday and Stein, 1987; Crenna and Frigo, 1987; Dietz, 2011). Reflex excitability between the legs are modulated out of phase during locomotion, and it is hard to predict if a significant correlation of bilateral reflex excitability will take place, as well as its functional role. Yet, the present investigation of common effects that disrupt a preexistent synchronism in reflex excitability can provide elements to better understand the neuronal organization responsible for coordinated motor actions between both sides.

### Leg Cycling and Bilateral Reflex Fluctuations in the Arms

The effects from rhythmic bilateral movement on reflexes of remote limbs are reciprocal. That is, leg cycling modulates H-reflexes in arm muscles in a manner similar to arm cycling on reflexes in the legs (Zehr et al., 2007; Nakajima et al., 2013). Here, with wrist flexor FCR at rest and without leg cycling, the amplitude of significant peak in the CCV at the zero-lag was higher than other conditions tested, probably due to tonic contraction from remote muscles as described in the previous section.

The amount of significant reduction in the amplitude of CCV peak at the zero-lag between both arms during leg cycling was not different than that observed during FCR voluntary contraction. The absence of interaction between “cycling” and “contraction” conditions indicates that both neuronal processes might be independent from each other. In fact, while rhythmic arm cycling leads to an increase in presynaptic inhibition at Ia afferent terminals from the FCR muscle (Nakajima et al., 2013), the voluntary tonic contraction has the opposite effect, i.e., it reduces presynaptic inhibition (Nielsen and Petersen, 1994). These effects, despite being opposite to each other, when combined (condition CyCo), induced a reduction of 68.1% in CCV amplitude from the St condition (without both cycling and contraction; see Figure 4). However, this amount of decrease did not represent the sum of both effects (cycling plus contraction: 89.9%), again, indicating no interaction between both processes. It can be reasoned that different neuronal processes are involved in the disruption of concomitant reflex fluctuations between the arms. However, at the present, it is not possible to conclude whether there is a superposition of the mechanisms or if they operate independently. Further experiments are necessary to clarify these questions.

During simultaneous rhythmic movement of all four limbs, there is a predominant influence of leg movements on the arms, i.e., an imposed cycling cadence to the legs decreased arm cycling cadence (Sakamoto et al., 2007). The “descending” effect was much weaker: arm cycling cadence had little or no effect on leg’s. A reduction in cadence of the arms might reflect a disruption in the coordination of bilateral movement. This can partially explain the present results, reduction in bilateral reflex excitability of the arms during both leg cycling and FCR voluntary contraction. Reduced concomitant reflex fluctuations could, therefore, be interpreted as a disruption in the action of commissural interneurons that convey neuronal commands to both sides. The dominance of these ascending effects from the legs was thought to be mediated by propriospinal interneurons that connect lumbar with cervical CPGs. As the CPGs from both sides of the spinal cord interact to each other via commissural interneurons (Shevtsova et al., 2015), these cells might change their excitability and, consequently, their activity. This in turn could contribute to changes in reflex synchronization reported here, reflecting an increased independence between the arms.

Additionally, propriospinal interneurons can be involved in bilateral coordinated action (Mitchell et al., 2016) and mediate the descending drive from supraspinal locomotor commands toward CPG networks within the spinal cord (Benthall et al., 2017). Evidence of a propriospinal pathway, conveying cortical commands to motoneuronal pools in upper limbs, has recently been provided in humans (Nakajima et al., 2017). These spinal cord elements could mirror the asymmetric excitability of both cortical hemispheres during voluntary bilateral contraction of FCR muscles (Pearce et al., 2005).

Arm movement during locomotion is an active neural process (Kuhtz-Buschbeck and Jing, 2012) to maintain coordinated actions with the legs (Zehr and Duysens, 2004). However, the neuronal coupling between the arms is weaker than that observed between the legs (Zehr et al., 2016). For instance, the effect of contralateral arm movement on H-reflex amplitude is reduced (Zehr et al., 2003) as compared to the same effect in the legs (Collins et al., 1993; Cheng et al., 1998; Mori et al., 2015). A similar weaker contralateral influence was found for cutaneous reflexes between the arms (Carroll et al., 2005). Therefore, the current results might be explained by the characteristic independent control of upper limbs in a vast repertoire of motor tasks, in contrast to postural muscles involved in locomotion (e.g., the SO muscle) that show a strong neuronal coupling related to propulsion during gait (Zehr et al., 2016).

### Methodological Considerations

As for the occurrence of significant peak at the zero-lag in the CCV sequence, an unexpected result was the high amplitude observed for both static (St) as compared to other conditions. An interesting fact, however, is the very similar mean amplitude of the CCV peak at the zero-lag form both arms and legs in St condition (compare the first column of the Figures 4A,B). According to our initial hypothesis, contraction and cycling should present higher amplitude in the CCV peak as compared to the St condition. However, it is important to emphasize that St condition in the present study is quite different than the reclining posture on a comfortable armchair from the subjects of Mezzarane and Kohn’s (2002) study. Here, the subjects had to hold the ergometer and muscle activation from arms was inevitable (see Figure 8). In addition, no support for the head was provided, resulting in tonic contraction of neck and probably the trunk muscles.

For reflex responses elicited in the FCR muscle, the SO muscles of both legs were quite silent in condition St, but the contraction of neck and trunk muscles probably was a constant influence throughout conditions.

The significant difference in the CV indicated that the relative variability of H-reflex was higher during cycling, regardless of muscle contraction or side. This holds true for both SO and FCR muscles (see Figure 7). In a seminal study on reflex variability in the cat, Rudomin and Dutton (1967) reported that increased level of presynaptic inhibition reduced spontaneous reflex fluctuations. The authors argued that presynaptic effects, rather than postsynaptic ones, are the main responsible for the reduction in reflex variability. If the suppression of the SO H-reflex from arm cycling is due to presynaptic mechanisms (Frigon et al., 2004), one should expect a reduction in reflex variability and not the opposite as currently observed. The higher relative variability found during cycling as compared to no-cycling condition could be ascribed to mechanical coupling within the limbs that would lead to instabilities during cycling. However, the low level of muscle activation suggests that such instabilities did not occur. Further investigations comparing the influence from rhythmic movements and induced changes in the level of presynaptic inhibition, e.g., by means of somatosensory conditioning stimulation technique (Mezzarane et al., 2012), must be conducted to clarify this issue.

## Conclusion

Our results extend and support other findings in the literature by showing significant suppressive effects on the SO H-reflex from arm cycling (Frigon et al., 2004; Loadman and Zehr, 2007; de Ruiter et al., 2010) and on the FCR H-reflex from leg cycling (Zehr et al., 2007; Nakajima et al., 2013). The increased H-reflex amplitude during voluntary contraction was also in agreement with earlier well-established reports (Crenna and Frigo, 1987; Schieppati, 1987; Funase and Miles, 1999; Misiaszek, 2003). Perhaps, one of the most striking results currently obtained was the absence of interaction between both tasks (contraction and cycling). This means that voluntary tonic muscle contraction did not affect the amount of suppression from cycling. For instance, the suppression of the SO H-reflex from arm cycling did not differ between relaxed and SO contraction conditions. This result suggests that the influence from neuronal oscillators from remote limb probably did not interact with the tonic drive from cortical motor regions. This speculation agrees with the CCV results showing that, despite the opposite effects on modulation of the H-reflex, both leg cycling and FCR contraction disrupted the synchronous reflex fluctuations within the arms. However, arm cycling did not change the reflex co-variability within the legs, while SO contraction did. This might represent an interesting perspective for the development of new analytical approaches in the studies of spinal cord neurophysiology. We suggest the current approach has an application in assessing changes in integrated neural connectivity between the limbs arising in acute or chronic neurological conditions such as Parkinson’s disease, multiple sclerosis and traumatic brain injury (Zehr et al., 2016).

## Author Contributions

RAM, EPZ and TN: conception or design of the work; acquisition, analysis, or interpretation of data for the work; revising it critically for important intellectual content; approved the final version of the manuscript; agree to be accountable for all aspects of the work in ensuring that questions related to the accuracy or integrity of any part of the work are appropriately investigated and resolved. RAM: drafting the work. All persons designated as authors qualify for authorship, and all those who qualify for authorship are listed.

## Conflict of Interest Statement

The authors declare that the research was conducted in the absence of any commercial or financial relationships that could be construed as a potential conflict of interest.
